# Exploring New Methods to Study and Moderate Proton Beam Damage for Multimodal Imaging on a Single Tissue Section

**DOI:** 10.1021/jasms.2c00226

**Published:** 2022-11-18

**Authors:** Catia Costa, Janella de Jesus, Chelsea Nikula, Teresa Murta, Geoffrey W. Grime, Vladimir Palitsin, Roger Webb, Richard J. A. Goodwin, Josephine Bunch, Melanie Jane Bailey

**Affiliations:** & University of Surrey Ion Beam Centre, Guildford, Surrey GU2 7XH, U.K.; ‡ Department of Chemistry, University of Surrey, Guildford, Surrey GU2 7XH, U.K.; § The National Physical Laboratory, Teddington, Middlesex TW11 0LW, U.K.; ∥ Imaging and Data Analytics, Clinical Pharmacology and Safety Science, R&D, AstraZeneca, Cambridge CB4 0WG, U.K.; ⊥ Institute of Infection, Immunity and Inflammation, College of Medical, Veterinary and Life Sciences, University of Glasgow, Glasgow G61 1QH, U.K.

## Abstract

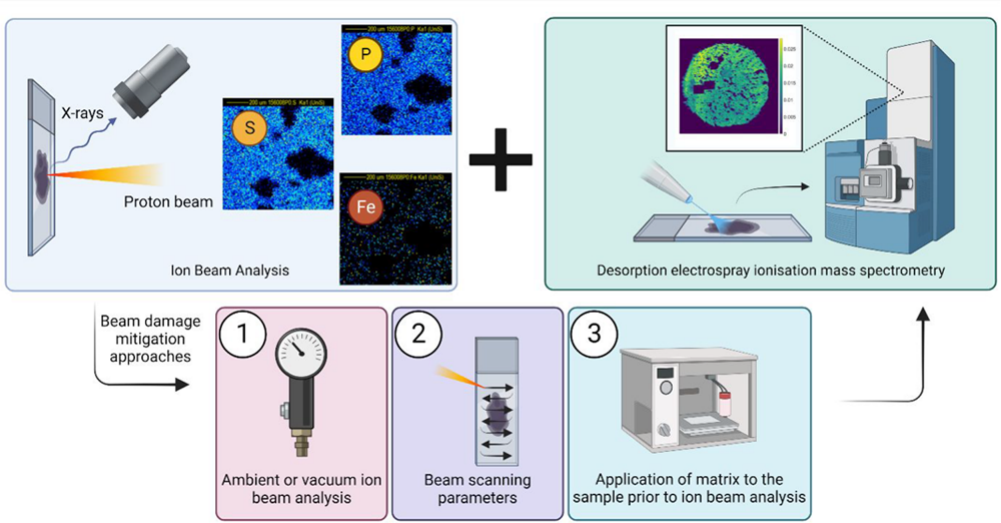

Characterizing proton beam damage in biological materials is of interest to enable the integration of proton microprobe elemental mapping techniques with other imaging modalities. It is also of relevance to obtain a deeper understanding of mechanical damage to lipids in tissues during proton beam cancer therapy. We have developed a novel strategy to characterize proton beam damage to lipids in biological tissues based on mass spectrometry imaging. This methodology is applied to characterize changes to lipids in tissues ex vivo, irradiated under different conditions designed to mitigate beam damage. This work shows that performing proton beam irradiation at ambient pressure, as well as including the application of an organic matrix prior to irradiation, can reduce damage to lipids in tissues. We also discovered that, irrespective of proton beam irradiation, placing a sample in a vacuum prior to desorption electrospray ionization imaging can enhance lipid signals, a conclusion that may be of future benefit to the mass spectrometry imaging community.

## Introduction

Multimodal imaging, in which multiple techniques are used to obtain complementary images, is of growing interest in various scientific disciplines—including forensics, cultural heritage, and biomedicine.^1^ In biomedicine, this approach is desirable to gain an enhanced understanding of disease pathogenesis and drug delivery or for biomarker discovery.^2^ Within this paradigm of analysis, the integration of imaging modalities that target both organic and elemental biomarkers is particularly pertinent, for example, to optimize the delivery of metal-containing drugs and nanoparticles or explore the impact of bioaccumulated metals on the host metabolome.^3,4^

To date, most studies considering both organic and elemental imaging have reported the use of different samples—for example, sequential sections of tissue. However, it is well established that smaller features in sequential tissue sections are not always accurately reproduced, particularly as imaging scales approach the single cell level.^5^ For this reason, methods that can be applied to the *same* tissue sample are highly desirable to enable the accurate colocation of elemental and molecular features. Recent work has shown that it is possible to carry out multimodal imaging of elemental and molecular markers on the same tissue sample by performing sequential desorption electrospray ionization (DESI) imaging, followed by elemental imaging using proton induced X-ray emission (PIXE).^3^ However, this method carries the disadvantage of the loss of the elements K and Cl following DESI analysis, presumably due to these mobile ions being desorbed by the water in the DESI spray solvent. Carrying out PIXE prior to DESI resolved this problem but resulted in a lower yield of detected lipids due to proton beam damage. We therefore consider here methods to reduce proton beam damage to lipids and metabolites in biological tissues to enable greater coverage of the metabolome and metallome from a single sample.

Previous work has considered proton beam damage to various sample types across multiple disciplines, for example, proton beam damage to graphene^6−8^ and polymers.^9−11^ Extensive research on proton beam damage to cultural heritage objects has also been carried out, but most studies considered only visible damage in paints, parchment, and paintings.^12−18^ Visible changes, as well as changes to light element concentrations in tissues and cells, have been observed following proton beam irradiation.^19,20^ The effect of proton beam irradiation to living cells has also been characterized in terms of biological response and DNA damage, due to the relevance to proton beam cancer therapy.^21,22^ There is, however, a dearth of information on proton beam damage to lipid or other metabolite markers in tissues, which this work seeks to address by considering mechanical damage to lipids.

It has been proposed^17^ that organic-based materials are the most susceptible to MeV ion beam induced changes due to their poorer resistance to electronic excitation or heating. As the proton beam travels through a material, the ion–electron interaction can lead to breakage of chemical bonds or formation of new ones, which will ultimately cause changes to the structure of the sample.^17^ There is general agreement that lowering the beam fluence reduces the risk of damage, but this concurrently causes a loss in PIXE performance.

PIXE imaging can either be performed under vacuum or in air. Ambient analysis normally results in slightly degraded spatial resolution, so it is less optimal for imaging but is known to reduce visible beam damage due to improved heat transfer in air.^23^ Similarly, increasing the scan speed has been shown to reduce visible damage to a frozen liver tissue section (10 μm thick) by reducing localized beam heating.^24^

Here we characterize proton beam damage to *molecular markers* in biological tissues for the first time, with the aim of recommending workflows for multimodal molecular and elemental imaging. We test three approaches designed to mitigate beam damage by reducing beam heating. First, we explore proton beam conditions that have been proposed to reduce visible damage, namely ambient analysis (method A)^25−27^ and variation in the scan rate (method B).^24,28^ The third method (method C) involves deposition of an organic matrix. Organic matrices are routinely used for matrix-assisted laser desorption ionization (MALDI), a mass spectrometry imaging technique. The matrix is deposited prior to laser irradiation and is used to absorb the laser energy and promote ionization of target analytes without fragmentation. The addition of matrix also increases the thermal conductivity of the sample.^29^ Liver homogenates were irradiated using a proton beam under each of the three conditions described above and sequentially analyzed with DESI. A peak list was generated through tentative assignment of *m*/*z* peaks detected in the samples and monitored to observe the effects of the different approaches tested here.

## Methods

### Sample Preparation

Liver homogenates were prepared as described by Swales et al.^30^ Liver tissue was homogenized and pipetted into molds (2 mL bottom end of Pasteur pipet bulb). The homogenates were snap frozen in liquid nitrogen and stored at −80 °C. The homogenates were sectioned into 10 μm thickness using a Thermo NX70 Cryostar (Thermo Scientific, Germany) and were thaw mounted onto polyethylene (PET) substrates (Leica, UK). Sectioned samples were vacuum packed and stored at −80 °C until analysis. Samples were brought to room temperature before being placed under vacuum for proton beam irradiation. All animals and tissue were managed in accordance with the UK Home Office Animals (Scientific Procedures) Act 1986. The organs used within this study were additionally used within the 3Rs principles as they comprise control material surplus to the original study for which they were intended.

#### Deposition of Organic Matrices

One investigated method for mitigating beam damage (method C) was to deposit organic matrix prior to irradiation. Three matrices were used: 2,5-dihydroxybenzoic acid (DHB), α-cyano-4-hydroxybenzoic acid (CHCA), and 9-aminoacridine (9-AA). Table S1, Supporting Information, details the preparation and concentration of the matrices used for this study. All matrices were deposited using an HTX-TM sprayer (HTX Technologies, USA) with a flow rate of 0.08 mL/min, 65 °C spray head temperature, 1333 mm/min track speed with 3 mm track spacing, a crisscross (CC) pattern, and 40 mm nozzle height. All matrices were applied to a nominal thickness of 1 μm using the number of passes described in Table S1.

### Ion Beam Analysis

Samples were irradiated at low, medium, and high fluence, nominally 5, 30, and 60 min measurement using a 2 MV Tandem accelerator (High Voltage Engineering, Netherlands). Table 1 documents the irradiation time, charge collected, and corresponding fluence for each experiment.

**Table 1:** Irradiation Time, Charge Collected, and Fluence for Each Proton Beam Irradiation Experiment

	Low Fluence	Medium Fluence	High Fluence
Vacuum	5 min, *Q* = 138 nC, Fluence = 8.63 × 10^22^ ions/cm^2^	30 min, *Q* = 818 nC, Fluence = 5.11 × 10^23^ ions/cm^2^	60 min, Q = 1650 nC, Fluence = 1.03 × 10^24^ ions/cm^2^
Ambient *vs* Vacuum (Method A)	5 min, Q = 109 nC, Fluence = 6.81 × 10^22^ ions/cm^2^	30 min, Q = 642 nC, Fluence = 4.01 × 10^23^ ions/cm^2^	60 min, Q = 1318 nC, Fluence = 8.24 × 10^23^ ions/cm^2^
Beam Scanning Parameters (Method B)	5 min, Q = 109 nC, Fluence = 6.81 × 10^22^ ions/cm^2^	30 min, Q = 642 nC, Fluence = 4.01 × 10^23^ ions/cm^2^	60 min, Q = 1318 nC, Fluence = 8.24 × 10^23^ ions/cm^2^
Organic Matrices (Method C)	5 min, Q = 91 nC, Fluence = 5.69 × 10^22^ ions/cm^2^	60 min, Q = 1074 nC, Fluence = 6.71 × 10^23^ ions/cm^2^	120 min, Q = 3234 nC, Fluence = 2.02 × 10^24^ ions/cm^2^

#### Ambient Pressure Irradiation

The proton beam was extracted into air through a 100 nm Si_3_N_4_ window, as described in Matjacic et al.^31^ The beam was focused to 9 × 9 μm, with a beam current of ∼300 pA and a scan size 0.75 × 0.75 mm. To compare ambient versus vacuum directly (method A), for a subset of samples, the beam spot size (9 × 9 μm) and current (300 pA) achieved in air was replicated in the vacuum chamber (see the next section) so that the irradiation conditions (fluence, flux, scan area, scan rate) were identical. For this experiment, a silicon drift detector (SDD) with a nominal energy resolution of 133 eV measured at the Mn Kα X-ray line at 5.9 keV (SGX Sensortech Ltd., U.K) was used to record the induced characteristic X-rays. The detector was equipped with a 12.5 μm DuraBeryllium entry window and placed at 28° from the surface normal. The active area of the detector was 25 mm^2^, and it was fitted with a 45 μm Kapton filter to prevent backscattered ions entering the detector. Helium gas was continuously supplied between the beam exit window and sample at a flow rate of 0.6 L/h.

#### Vacuum Pressure (10^–6^ mBar) Irradiation

Samples were placed in a vacuum chamber pumped to 10^–6^ mBar and irradiated using 2.5 MeV protons with beam currents ranging from 300 to 600 pA. The beam was focused to approximately 2 × 2 μm (measured using a 75 × 75 μm 1000 copper grid). The scan size was 1 × 1 mm. X-rays were detected using a SDD fitted with a 130 μm Be filter, mounted at an angle of 135° to the beam direction in the horizontal plane. Backscattered particles were simultaneously collected and detected using a passivated implanted planar silicon (PIPS) detector with an active area of 150 mm^2^, placed 52.5 mm away from the sample and mounted at 25° exit angle.

For standard PIXE imaging experiments, the pixel dwell time was set at 0.3 ms and the beam was scanned in a sawtooth raster pattern. To explore the effect of proton beam scan speed and pattern on beam damage (method B), scan parameters were changed as follows: pixel dwell time was changed to 0.03 ms (slow scanning) or 3 ms (fast), while keeping the sawtooth raster pattern, and the raster pattern was changed to “random”, while maintaining a 0.3 ms pixel dwell time.

#### Data Dnalysis: IBA

The X-ray and backscattered particle spectra were calibrated using a BCR-126A lead glass standard. Data was acquired and analyzed using OMDAQ-3 software (Oxford Microbeams, Ltd. UK).^32^

### DESI Imaging

DESI was used to image molecular markers in the tissue homogenates after ion beam irradiation. These images were used to monitor the effectiveness of different methods for mitigating irradiation effects. A prototype DESI source with a recessed capillary (Waters, UK) was coupled to a Xevo G2-XS (Waters, UK) mass spectrometer. A 95:5 (%v/v) methanol/water spray solvent was delivered at 2 μL/min using an Ultimate 3000 UHPLC system (Thermo Fisher, Germany) with an electrospray voltage of 0.6 kV and ion block temperature set to 100 °C. Prior to acquisition, mass calibration in positive-ion mode was performed using a film of polylactic acid (PLA) deposited on a slide by sublimation (made in house), with a collision energy of 35 V. Data were acquired in positive ion “sensitivity” mode, with a mass range of *m*/*z* 100–1200 at a calculated mass resolving power of 15000 at *m*/*z* 200. The tissue region for imaging was selected using HDI Imaging (Waters, UK). The nominal pixel size was 75 × 75 μm using a stage speed of 150 μm/sec, acquiring the data at 2 pixels/s.

#### Data Analysis: DESI

Waters RAW data files were converted into imzML files through a two-step conversion. The first is the conversion to mzML using Proteowizard^33^ and then to an imzML using an imzML converter.^34^ The imzML data was analyzed using Spectral Analysis^35^ (version 1.4.0), run using MATLAB (version 2018b). Prior to generating a mean spectrum, data were preprocessed using a rebinning method (bin size of 0.001) to generate the mean spectra and then normalized to the total ion intensity when generating the datacube.

A putative lipid feature peak list was generated through tentative of assignment of *m*/*z* peaks detected in the liver homogenates, using in-house MATLAB scripts which matched the data against the Human Metabolome Database (HMDB).^36^ Peak assignment was achieved using a ±15 ppm mass match and through inspection of the DESI ion images to ensure that the signals originated from the sample and not the background. The peak list was monitored for all experiments and comprised lipids from the following classes: diacylglycerols (DAG), fatty acids (FA), lysophosphatidylcholines (LPC), lysophosphatidylethanolamines (LPE), phosphatidic acids (PA), phosphatidylcholines (PC), phosphatidylethanolamines (PE), and phosphatidylglycerol (PG) (see Table S2, Supporting Information).

## Results

Homogenized liver tissue was used as a model sample to enable comparison of the different methods to mitigate beam damage.

**Figure 1:**
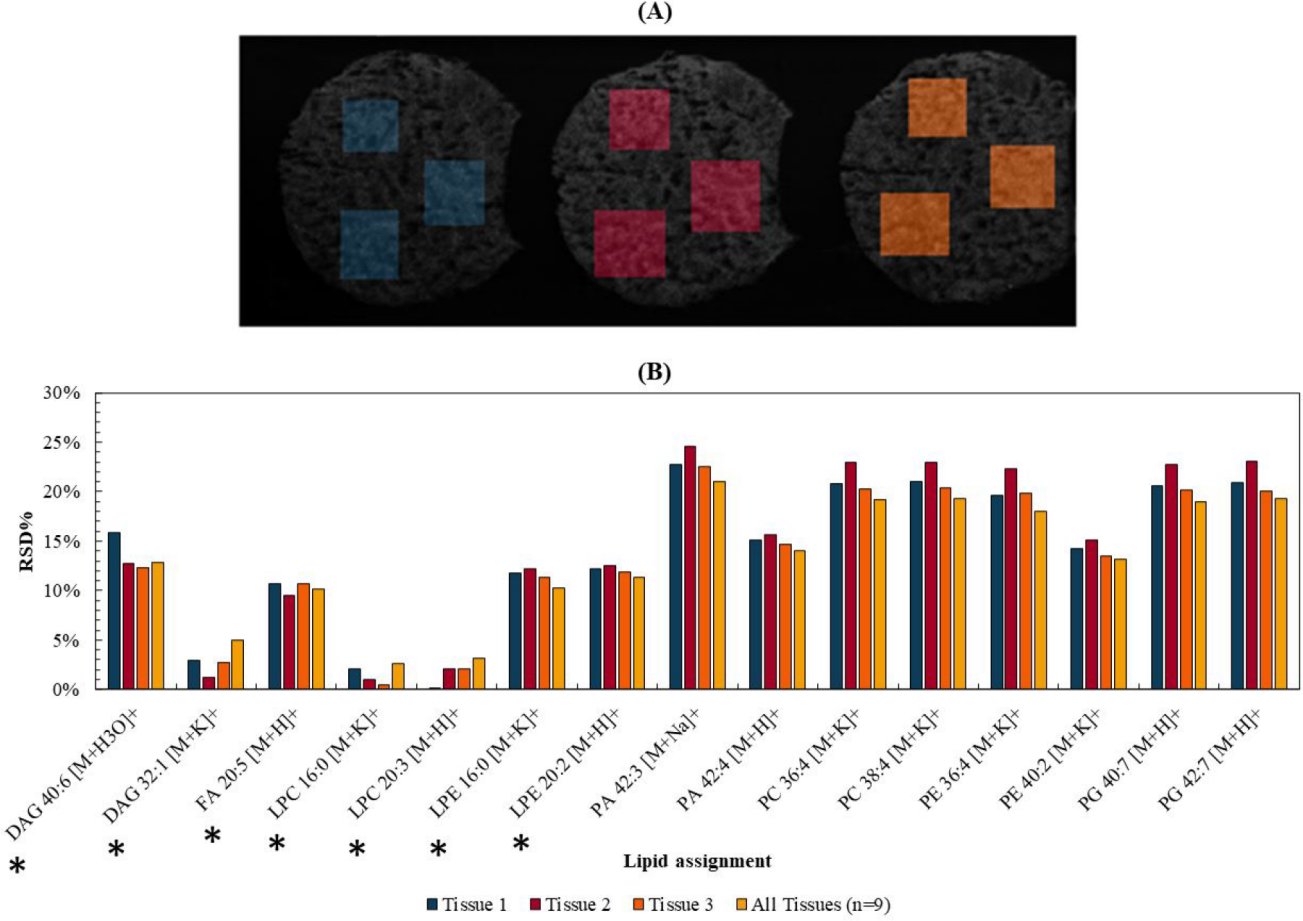
(A) Total ion count (TIC) image for the three sequential tissue sections and respective regions of interest (ROI) selected. (B) Relative standard deviations (RSD %) of normalized (to TIC) data of three ROIs taken on three tissue homogenates and across all three sections (*n* = 9). *Indicates compounds with *m*/*z* < 700.

First, we characterized the *intra*- and *inter*-sample variability and the repeatability of the DESI. Three consecutive tissue sections were analyzed using DESI, and in each section, three regions of interest (ROI) were selected (see Figure 1(A)). Figure 1(B) shows the variability in normalized peak intensity between the different ROIs for different analytes. For all analytes, the *intra*- and *inter*-sample variability was below 25%. For compounds below *m*/*z* 700 (highlighted with an asterisk in Figure 1(B)), the variability across all three tissues is lower than 16%, increasing to 20–25% for *m*/*z* > 700. This is consistent with previous studies that have tested the repeatability of DESI.^37−39^Figure 1(B) demonstrates the homogeneity within and between the tissue sections, as well as the repeatability of the DESI analysis.

**Figure 2:**
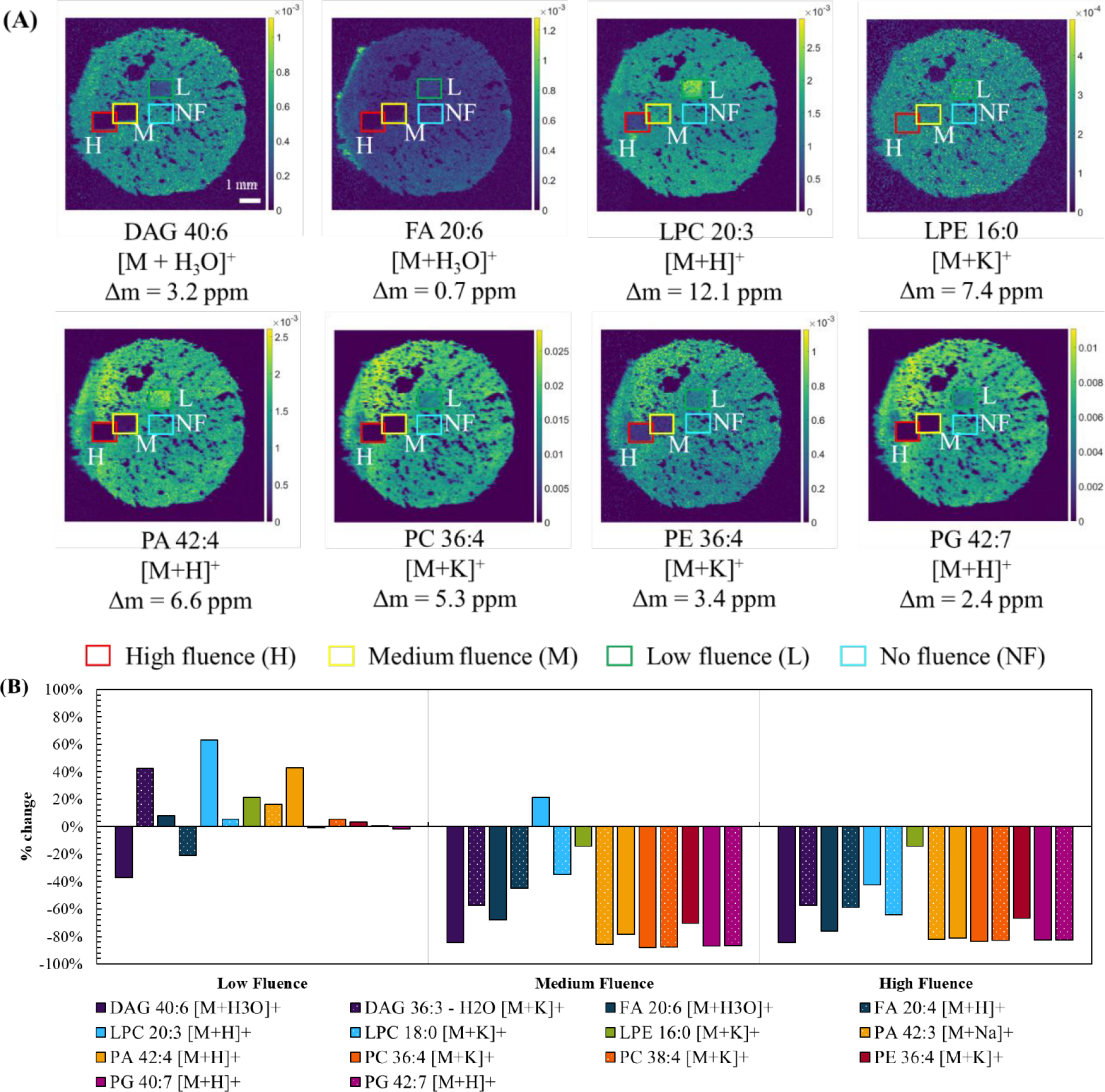
(A) Extracted ion maps obtained using DESI following irradiation with a 2.5 MeV proton beam under vacuum, irradiated at % low, medium, and high fluences as described in Table 1. (B) Percentage change (% change) in TIC-normalized peak intensity between a low, medium, or high fluence area and a nonirradiated area (no fluence).

Next, we looked at the baseline changes to lipid profiles after proton beam irradiation with a 2.5 MeV proton beam at three different fluences under vacuum (see Table 1). Figure 2(A) shows DESI images of a selection of *m*/*z* values from the peak list. The highlighted areas show the irradiated ROIs, and Figure S1 shows the ROIs selected for data processing. These fluences correspond to the typical laboratory conditions used to detect major (low fluence), minor (medium fluence), and trace elements (high fluence) in tissues (see Table 1). Figure 2(B) shows the percentage change in normalized signal intensity between these ROIs and a nonirradiated region on the same sample. At low fluence, some lipids (e.g., peaks assigned to PA and PE lipids) present an increase in peak intensity in the proton beam irradiated areas. This may be due to ionization enhancement of these species arising from changes in surface chemistry or because these lipids are fragments derived from larger molecules which were fragmented during ion beam irradiation. In contrast, other species (e.g., PG and PC lipids) show little change. At medium and high fluences, there is a reduction in peak intensity for most lipid species. The ion maps in Figure 2(A) clearly highlight the need to reduce proton beam damage to lipids in tissues to enable subsequent mass spectrometry imaging. This is because high fluences are required to image trace elements, and Figure 2 shows that this leads to reduced sensitivity to analytes subsequently targeted by mass spectrometry imaging (MSI).

Three methods were investigated to determine whether proton beam irradiation effects could be reduced. They were performing irradiation at ambient pressure (external beam) rather than in vacuo to improve thermal transfer (Method A), varying the beam scan speed to reduce localized beam heating (Method B), and adding an organic matrix prior to irradiation to increase the thermal conductivity of the tissue (Method C).

### Method A: Ambient Pressure vs Vacuum Pressure (10^–6^ mBar) Irradiation

**Figure 3:**
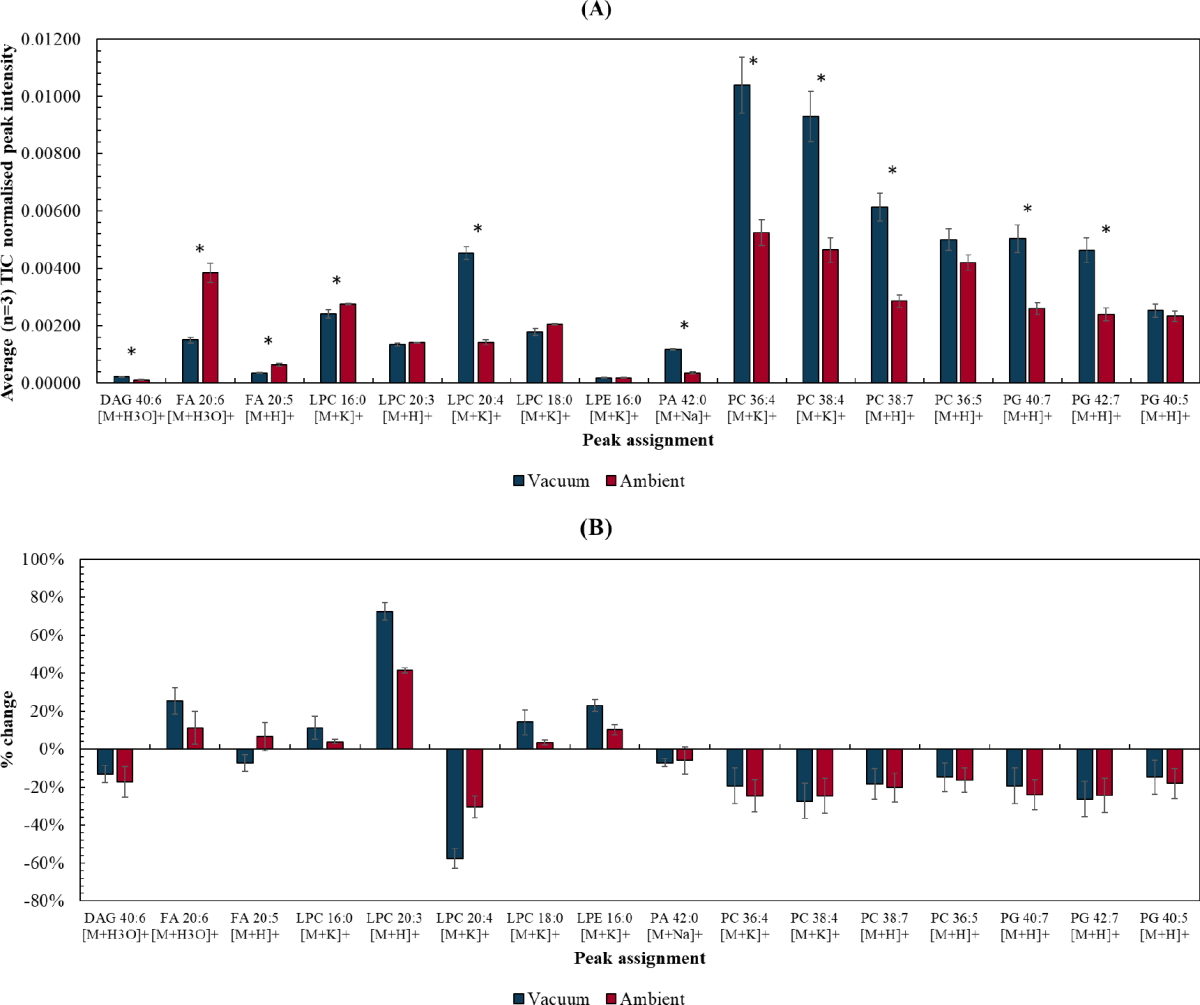
(A) Average (*n* = 3) normalized (to TIC) peak intensity for a range of lipid species taken from unirradiated regions of interest (approximately 1 × 1 mm areas) of tissue sections irradiated under ambient or vacuum conditions and sequentially imaged using DESI. * is used to show differences with *p* < 0.05 (Table S3, Supporting Information). (B) % change in normalized (to TIC) peak intensity between “low fluence” and “no irradiation” ROIs on tissue sections irradiated under ambient or vacuum conditions. Error bars represent the RSD% of the normalized peak intensities taken across the 3 regions of interest.

Sequential sections of homogenized tissue were proton beam irradiated under vacuum and under ambient pressure, respectively. The irradiated tissues were then imaged using DESI. First, lipid signals in unirradiated areas of the tissue were compared, to explore whether placing tissue in vacuum has any effect on sensitivity. Figure 3(A) shows that PA, DAG, and some PC, PG, and LPC lipid peaks were higher in the sample submitted to vacuum, while FA peaks were higher in the sample kept under ambient pressure. Overall, 8 of the 16 lipids detected across both samples presented higher (*p* < 0.05) peak intensities in the *vacuum sample*, suggesting that submitting the sample to vacuum is not problematic for sequential mass spectrometry imaging experiments and may even help to enhance detection of these analytes.

Figure 3(B) shows the percentage change between the “low fluence” and “no irradiation” ROIs in each tissue sections irradiated under vacuum or ambient conditions (see Figure S3, Supporting Information for ROI selection). The FA, LPE, and LPC lipids indeed showed less change after irradiation in ambient, suggesting that ambient irradiation does indeed have a protective effect. However, for PG, PC, and PA, irradiation under ambient pressure did not appear to mitigate beam damage. Figure S2, Supporting Information, shows the percentage change between the “medium” and “high” fluence and “no irradiation” ROIs irradiated under vacuum or ambient conditions. At medium fluence, the % change observed after vacuum irradiations is generally higher than that for the sample irradiated under ambient pressure. However, at high fluence, the damage observed is similar for both conditions. This suggests that irradiation at ambient pressure does offer some cooling effect, but only up to a certain fluence. Figure S3, Supporting Information, shows the extracted ion maps for some of the tentatively assigned lipids below, emphasizing the irradiated regions.

### Method B: Proton Beam Scan Speed/Pattern

Reports in the literature described how the visible damage caused by a high energy beam on a biological sample can be minimized by high speed scanning.^24,28^ Here we scan the proton beam at three different scan speeds (fast, standard and slow) and in a random pattern to observe whether this leads to differences in the damage to lipids, observed by subsequent DESI measurements.

Figure S4 shows the resulting PIXE maps for phosphorus (P), sulfur (S), chlorine (Cl), potassium (K), and iron (Fe) at the different scan speeds and pattern. The changes in scan speed did not affect the resulting PIXE maps. In the random scanning mode, pixels are collected in a random order, therefore distributing the heat load more evenly over the sample. This approach does not produce PIXE images but was used explore the effect of heat distribution on lipid profiles.

**Figure 4:**
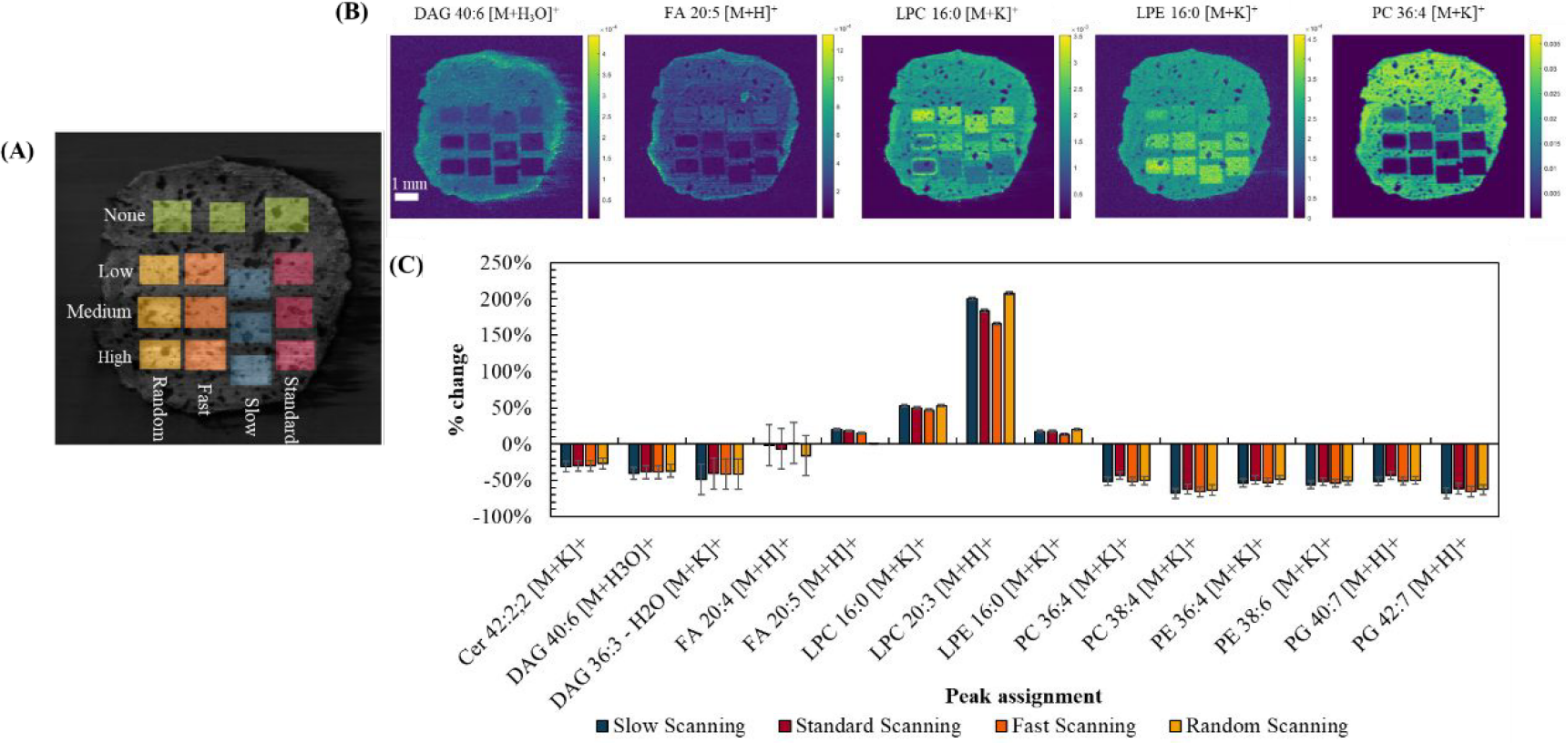
(A) ROIs for irradiated and nonirradiated areas. (B) DESI ion maps of selected lipid peaks showing the irradiated areas. (C) % change between the “low fluence” and no irradiation areas at different scan speeds and pattern.

Figure 4(B) shows the resulting DESI maps for a selection of lipid features, presenting the irradiated areas. Figure 4(C) shows the percentage change between the investigated scan speeds and scan pattern and a control (nonirradiated ROI). The changes observed using DESI for each of the beam scan speeds/pattern were similar for a given fluence, suggesting that this is not a worthwhile option to mitigate any of the irradiation effects on lipid metabolites and was therefore not considered further.

### Method C: Addition of Organic Matrix

Three different matrices (DHB, CHCA, and 9-AA) were tested and compared to a control (no matrix) for their ability to reduce proton beam damage. No changes to the spectra or images were detected by PIXE after matrix deposition, demonstrating that prior matrix addition does not adversely affect PIXE imaging (Figure S5(A) and Figure S6, Supporting Information). The addition of each matrix is detectable in the backscattered particle spectra (Figure S5(B–E), Supporting Information), which are collected simultaneously with PIXE to measure major elements (carbon, nitrogen, and oxygen). Fitting of the backscattered particle spectra allows X-ray absorption by the sample matrix to be calculated, enabling standard-free quantitative elemental analysis—a significant advantage of PIXE over other X-ray spectroscopy methods. Deposition of matrix did not adversely affect the ability to fit the backscattered particle spectra.

**Figure 5:**
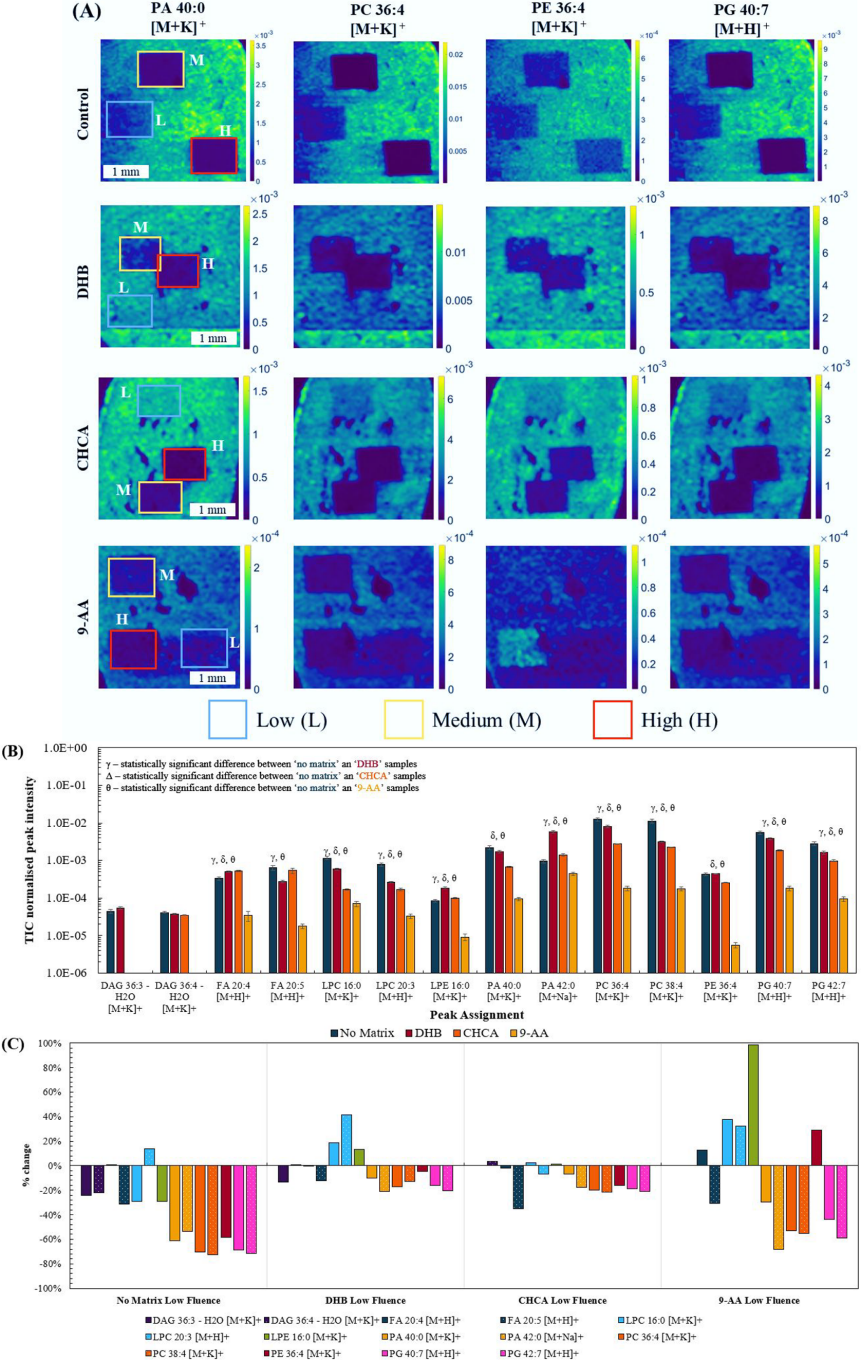
(A) Extracted ion maps of lipid signals obtained using DESI of tissue homogenates coated with matrices (DHB, CHCA, 9-AA) and a control (no matrix) after proton beam irradiation. (B) Normalized (to TIC) peak intensity taken from nonirradiated areas (*n* = 3) in each of the tissue sections. Lipids marked with an γ, δ, and θ present statistically different levels (*p* < 0.05) between the control and DHB, control, and CHCA and control and 9-AA, respectively (see Table 4, Supporting Information). (C) Percentage change between low fluence and no fluence ROIs in the same sample.

Extracted ion images are shown in Figure 5(A) for a selection of lipid peaks. Figure 5(B) shows the normalized peak intensity measured from nonirradiated ROIs in each of the tissue sections (see Figure S7, Supporting Information, for selected ROIs). Lower peak intensities were observed after deposition of 9-AA, which may negatively impact the sensitivity of the DESI experiment. This is not unexpected given that 9-AA is often used for MALDI experiments in negative-ion mode. Deposition of DHB and CHCA matrices produced peak intensities that were closer to the control. Figure S8 shows the overlay of spectra taken with DESI of tissue section regions (nonirradiated) coated with the different matrices, showing broadly similar profiles.

Figure 5(C) shows the % change between the low fluence and nonirradiated ROIs in the same sample. Addition of matrix did mitigate some of the damage when compared to the control sample, with DHB showing % changes closer to zero for most lipids. CHCA also provided some protection for a more limited set of peaks. This result is confirmed by Figure 5(A), where in the control sample there is a significant loss of intensity in the images, even after low fluence irradiation. However, after application of DHB or CHCA matrices, low fluence irradiation has little impact on the image intensity, and some signal is still visible even after medium and high fluence irradiation. Figure S9, Supporting Information, shows the % change between the “medium” or “high” fluence ROIs and the nonirradiated ROI.

## Discussion

This work has demonstrated that DESI imaging of proton-irradiated areas is a suitable methodology for the exploration of proton beam damage. This is because the observed changes to tissues are greater than the measurement precision and inter-/intrasample variability. The data shows that proton beam irradiation creates significant changes to lipid metabolites in tissues, increasing with higher fluence. These must be mitigated if PIXE is to be performed prior to mass spectrometry imaging (e.g., DESI, MALDI, SIMS).

**Figure 6:**
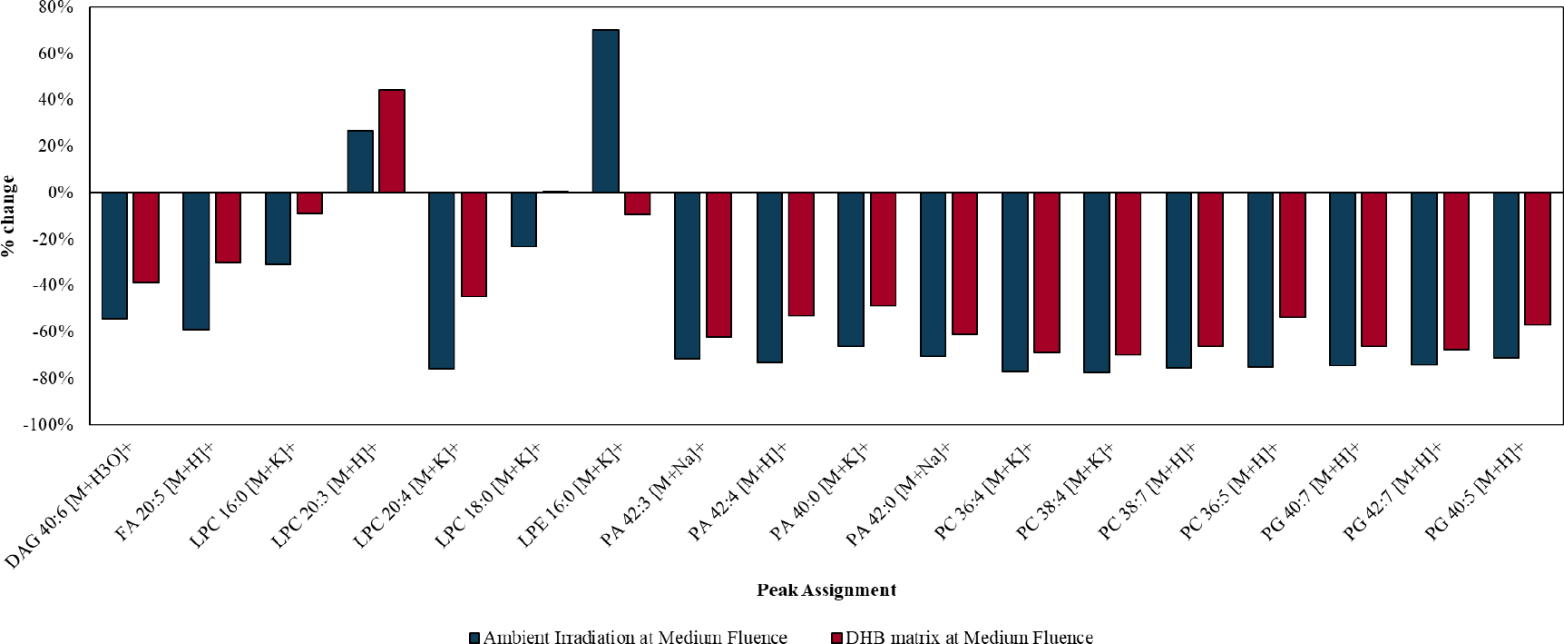
Percentage change between medium fluence and no fluence in the “ambient irradiation” and “irradiation in the presence of DHB matrix” experiments.

Deposition of an organic matrix was found to provide the greatest mitigation of beam damage, as shown in Figure 6 which compares the percentage changes at medium fluence for the ambient irradiation and irradiation in the presence of DHB matrix experiments. This was further confirmed by a *t* test which showed that the results of the two experiments were significantly different (*p* < 0.05). Application of DHB provided no elemental artifacts and allowed imaging of lipid species to take place at a higher fluence. We propose that the reason for this observation is that the DHB increases the thermal conductivity of the sample, thereby reducing beam heating during ion beam analysis.

Many ion beam analysis facilities use vacuum chambers for analysis because the analytical performance is superior. This work shows that submitting samples to vacuum is not problematic for subsequent mass spectrometry imaging and, in fact, may result in some sensitivity gains. Whether or not this works on all tissue types and the exact vacuum conditions needed for signal enhancement is beyond the scope of this work but should be explored in future studies.

In previous work, it has been reported that performing DESI prior to PIXE imaging results in delocalization of mobile ions, e.g., Cl and K.^3^ Therefore the proposed workflow could be used to study the local biochemistry in the presence of Cl or K accumulation to shed new insight into the impact of these elements on lipid metabolism, for example. However, it should be noted that even with damage mitigation this new workflow still results in a ∼60% loss of peak intensity, meaning that the sensitivity to lipids will be reduced.

In future work, DESI imaging could be applied to study the effect of proton beam irradiation to living tissues. Such a study may give insight into the metabolic pathways that are disrupted following proton beam irradiation. This study has only sampled ex vivo tissues and therefore only reveals the mechanical and heat induced damage caused by a proton beam, rather than mechanistic effects.

## Conclusions

Irradiation effects using a MeV energy beam present a challenge for multimodal imaging using the same tissue sample. We have explored three different methods aimed at mitigating proton beam damage—performing analysis under ambient conditions, changing the beam scan speed and pattern, and adding an organic matrix. Of the conditions tested, deposition of DHB matrix was found to provide the greatest protective effect, with ambient analysis providing some limited protection against proton beam damage.

We found that submitting a tissue sample to vacuum increased the signal intensity of most lipid species monitored. This suggests that exposing tissues to vacuum prior to analysis provides sensitivity gains for mass spectrometry imaging experiments.

## Supporting Information Available

(Table S1) Matrix preparation information; (Table S2) List of lipid features and respective *m*/*z* values; (Figure S1) ROIs selected for irradiation and data analysis in the damage example experiment; (Table S3) *t* test results for ambient versus vacuum experiments; (Figure S2) % change of lipid peak intensity after medium and high fluence for vacuum and ambient irradiations; (Figure S3) DESI ion maps after vacuum and ambient irradiations and extra experimental information on irradiation areas for vacuum versus ambient experiments; (Figure S4) PIXE maps taken at different scan speeds and pattern; (Figure S5) Overlay of X-ray spectra and fitted EBS spectra taken from homogenates coated with matrix; (Figure S6) PIXE maps taken from liver homogenates in the presence of different matrices and fluences; (Figure S7) Extra experimental information on irradiation areas for matrix-coated samples; (Figure S8) Overlay of DESI spectra taken from liver homogenates coated with different matrices; (Figure S9) % change of lipid peak intensity after medium and high fluence in the presence of matrices; (Table S4) *t* test results for matrix experiments; (Table S5) *t* test results comparing the medium fluence irradiations for the “ambient” and “DHB” experiments (PDF)
