# Supplementary material for: Exploring New Methods to Study and Moderate Proton Beam Damage for Multimodal Imaging on a Single Tissue Section
Source: J Am Soc Mass Spectrom. 2022 Nov 18;33(12):2263–72. doi: 10.1021/jasms.2c00226 (PMC9732869; doi:10.1021/jasms.2c00226)
Supplement: Supplementary file 1 — js2c00226_si_001.pdf [file js2c00226_si_001.pdf]

Exploring new methods to study and moderate proton beam damage for multimodal imaging on a single tissue section

Catia Costa[a¥], Janella de Jesus[b,c¥], Chelsea Nikula[c], Teresa Murta[c], Geoffrey W. Grime[a], Vladimir Palitsin[a], Roger Webb[a], Richard J. A. Goodwin[d,e], Josephine Bunch[c], Melanie Jane Bailey[a,b,*]

[a] University of Surrey Ion Beam Centre, Guildford, Surrey GU2 7XH, UK; [b] Department of Chemistry, University of Surrey, Guildford, Surrey GU2 7XH, UK; [c] The National Physical Laboratory, Teddington, Middlesex TW11 0LW, UK; [d] Imaging and Data Analytics, Clinical Pharmacology and Safety Science, R&D, AstraZeneca, Cambridge, CB4 0WG, UK; [e] Institute of Infection, Immunity and Inflammation, College of Medical, Veterinary and Life Sciences, University of Glasgow, Glasgow, G61 1QH, UK

[¥] Joint first authors

Address reprint requests to Melanie J. Bailey, Department of Chemistry, University of Surrey, Guildford, Surrey GU2 7XH, UK, +44 (0)1483682593. m.bailey@surrey.ac.uk

Table S 1. Preparation of matrices.

Matrix	Concentration	# of passes
DHB	37.5 mg/mL in 70% EtOH	3
CHCA	5 mg/mL in 80% MeOH	17
9-AA	10 mg/mL in 50% MeOH	8

Table S 2. List of 32 tentative lipid assignments and their theoretical m/z. Peak matching was performed based on +/- 15 ppm mass deviation.

Theoretical *m/z*	Tentative Lipid Assignment
**687.556**	DAG 40:6 $[M+H_3O]^+$
**605.454**	DAG 32:1 $[M+K]^+$
**639.475**	DAG 36:3 - H_2O $[M+K]^+$
**637.459**	DAG 36:4 - H_2O $[M+K]^+$
**319.227**	FA 20:6 $[M+H_3O]^+$
**305.248**	FA 20:4 $[M+H]^+$
**303.232**	FA 20:5 $[M+H]^+$
**534.296**	LPC 16:0 $[M+K]^+$
**546.355**	LPC 20:3 $[M+H]^+$
**582.296**	LPC 20:4 $[M+K]^+$
**562.327**	LPC 18:0 $[M+K]^+$
**492.249**	LPE 16:0 $[M+K]^+$
**470.288**	LPE 16:1 [M+H3O] $^+$
**498.259**	LPE 18:3 $[M+Na]^+$
**506.324**	LPE 20:2 $[M+H]^+$
**805.572**	PA 42:3 $[M+Na]^+$
**781.574**	PA 42:4 $[M+H]^+$
**799.561**	PA 40:0 $[M+K]^+$
**811.619**	PA 42:0 $[M+Na]^+$

**820.525**	PC 36:4 [M+K] $^{+}$
**848.557**	PC 38:4 [M+K] $^{+}$
**804.554**	PC 38:7 [M+H] $^{+}$
**780.554**	PC 36:5 [M+H] $^{+}$
**778.478**	PE 36:4 [M+K] $^{+}$
**838.572**	PE 40:2 [M+K] $^{+}$
**802.478**	PE 38:6 [M+K] $^{+}$
**768.554**	PE 38:4 [M+H] $^{+}$
**821.533**	PG 40:7 [M+H] $^{+}$
**849.564**	PG 42:7 [M+H] $^{+}$
**797.530**	PG 36:2 [M+Na] $^{+}$
**825.564**	PG 40:5 [M+H] $^{+}$

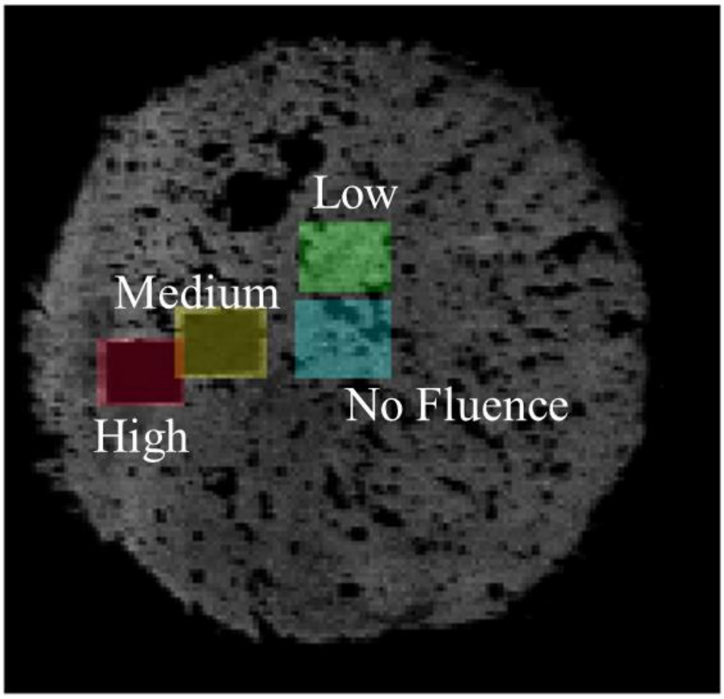

Figure S 1. Irradiated areas and non-irradiated areas (selected for data analysis) in liver tissue homogenates irradiated with a 2.5 MeV proton beam under vacuum, irradiated at %low, medium and high fluences.

Table S 3. T-test results for the different lipid peaks observed in tissue sections irradiated under vacuum and ambient pressure.

	Discovery?	P value	Mean of Vacuum	Mean of Ambient	Difference	SE of difference	t ratio	df	q value
**DAG 40:6 [M+H_3O]$^+$**	Yes	0.000255	0.00021	0.00011	0.0001	8.17E-06	12.25	4	0.000484
**FA 20:6 [M+H_3O]$^+$**	Yes	0.00032	0.001493	0.003847	-0.002353	0.0002035	11.56	4	0.000484
**FA 20:6 [M+H_3O]$^+$**	Yes	0.000607	0.0003567	0.00063	-0.0002733	2.79E-05	9.801	4	0.000614
**LPC 16:0 [M+K]$^+$**	Yes	0.018032	0.00241	0.002747	-0.0003367	8.71E-05	3.867	4	0.009934
**LPC 20:3 [M+H]$^+$**	No	0.152484	0.001343	0.001407	-6.33E-05	3.59E-05	1.764	4	0.066004
**LPC 20:4 [M+K]$^+$**	Yes	0.000025	0.004533	0.001417	0.003117	0.0001408	22.14	4	0.000075
**LPC 18:0 [M+K]$^+$**	No	0.022295	0.001783	0.002037	-0.0002533	6.99E-05	3.623	4	0.011259
**LPE 16:0 [M+K]$^+$**	No	>0.999999	0.0001767	0.0001767	0	4.71E-06	0	4	0.37875
**PA 42:0 [M+Na]$^+$**	Yes	0.000002	0.001177	0.0003567	0.00082	2.06E-05	39.91	4	0.000014
**PC 36:4 [M+K]$^+$**	Yes	0.001165	0.01038	0.005247	0.005133	0.0006205	8.273	4	0.000804
**PC 38:4 [M+K]$^+$**	Yes	0.001143	0.009297	0.00464	0.004657	0.0005601	8.314	4	0.000804
**PC 38:7 [M+H]$^+$**	Yes	0.000468	0.006133	0.002857	0.003277	0.0003126	10.48	4	0.000568
**PC 36:5 [M+H]$^+$**	No	0.040386	0.005	0.004193	0.0008067	0.0002699	2.989	4	0.018826
**PG 40:7 [M+H]$^+$**	Yes	0.001251	0.00504	0.002593	0.002447	0.0003013	8.121	4	0.000804
**PG 42:7 [M+H]$^+$**	Yes	0.001327	0.004627	0.0024	0.002227	0.0002785	7.994	4	0.000804
**PG 40:5 [M+H]$^+$**	No	0.310569	0.00252	0.002327	0.0001933	0.0001667	1.16	4	0.12547

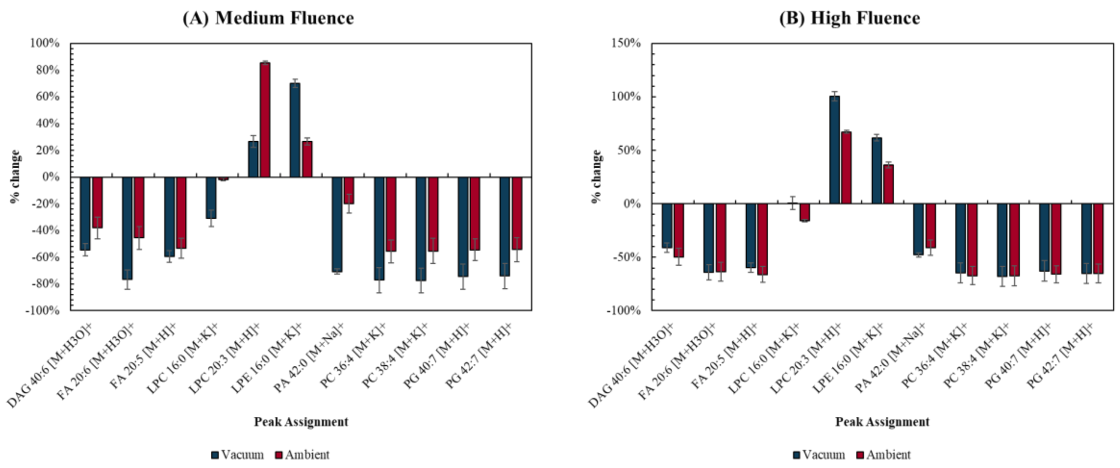

Figure S 2. % change in normalised (to TIC) peak intensity between 'medium' fluence (A) or 'high' fluence (B) and 'no fluence' irradiation ROIs on tissue sections irradiated under ambient or vacuum conditions. Error bars represent 1 standard deviation of the normalised peak intensities taken across the 3 regions of interest in Fig. 3 (A).

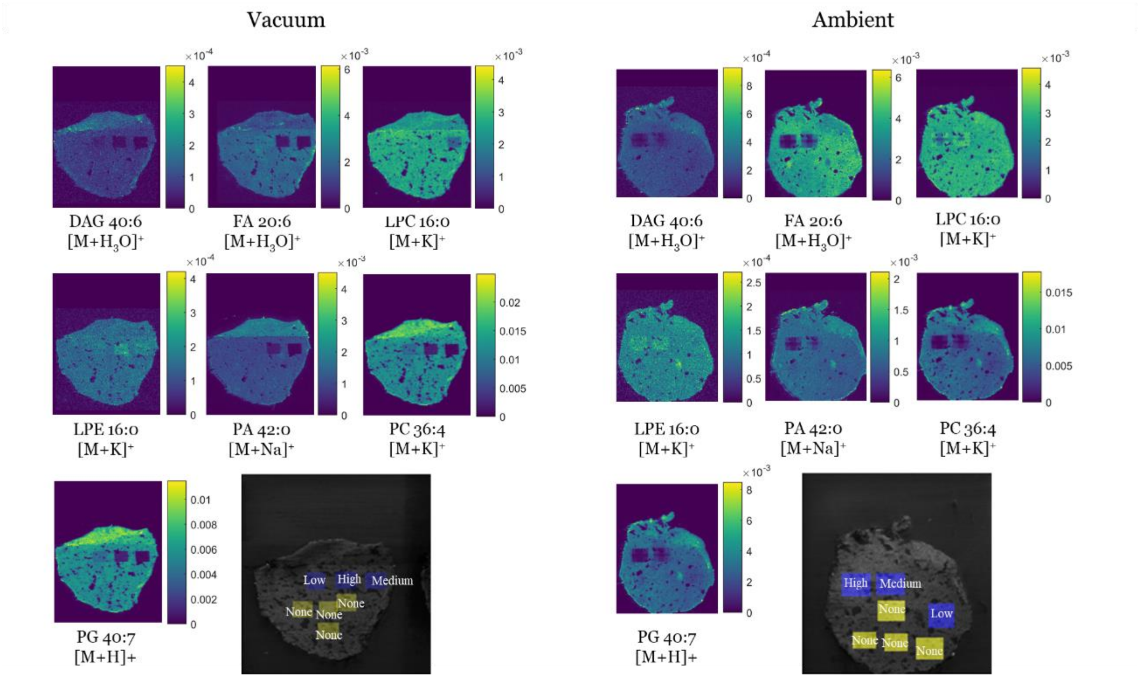

Figure S 3. Extracted ion maps of several tentatively assigned lipids (see Supplemental Data Table 1) obtained using DESI of tissue homogenates previously irradiated under vacuum (left) or ambient (right) conditions, showing the irradiated locations.

Element	Scanning Conditions	Low Fluence	Medium Fluence	High Fluence
Phosphorus (P)	Slow Scanning	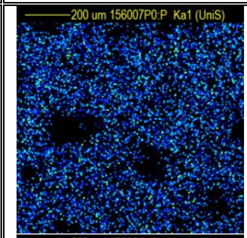	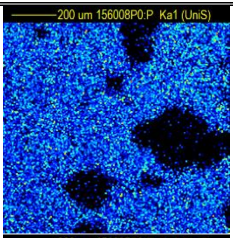	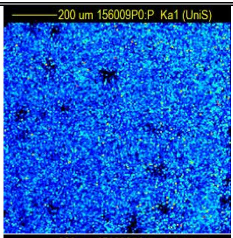
	Standard Scanning	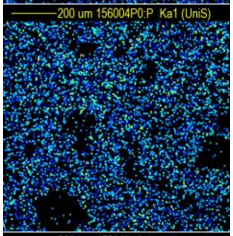	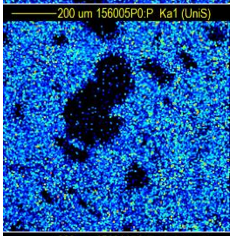	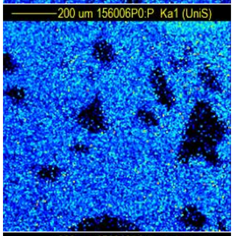
	Fast Scanning	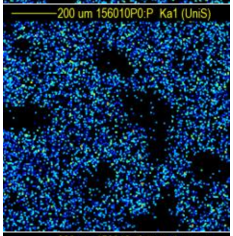	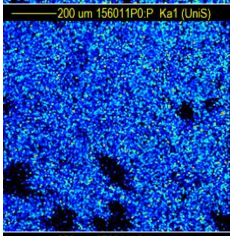	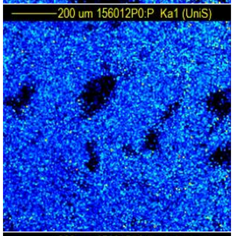
	Random Scanning	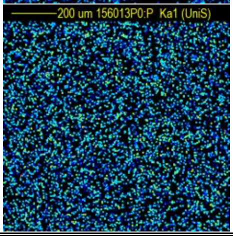	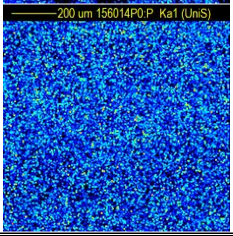	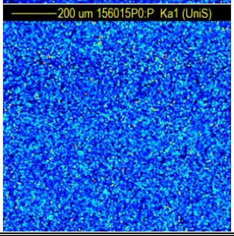
Sulphur (S)	Slow Scanning	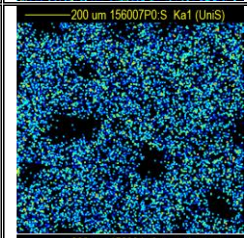	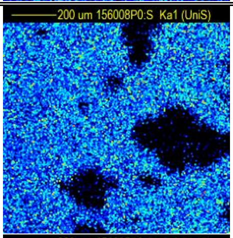	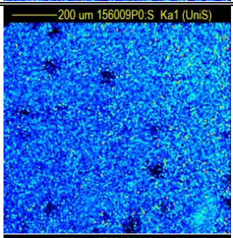
	Standard Scanning	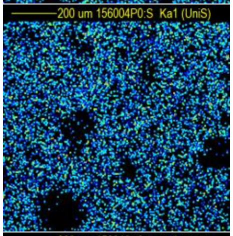	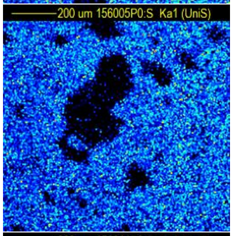	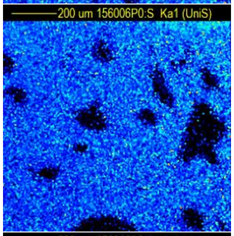
	Fast Scanning	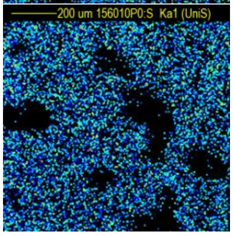	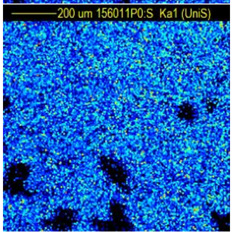	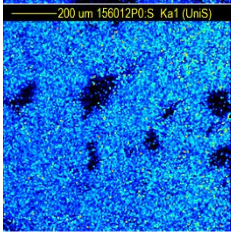

	**Random Scanning**	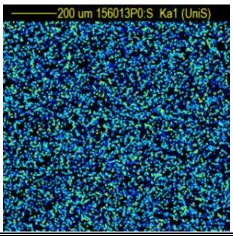	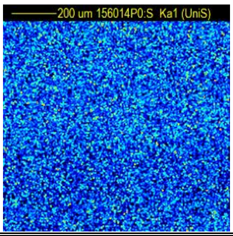	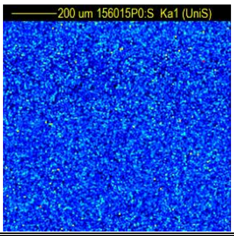
**Chlorine (Cl)**	**Slow Scanning**	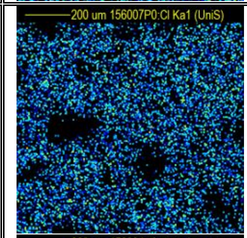	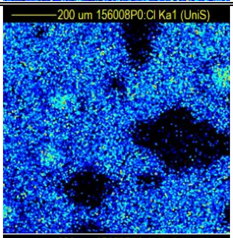	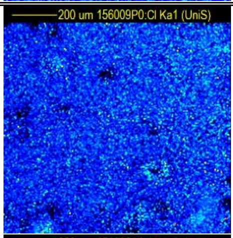
	**Standard Scanning**	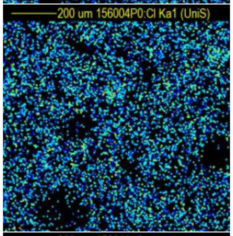	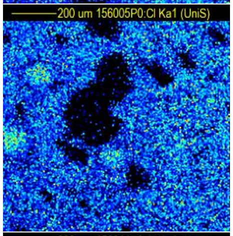	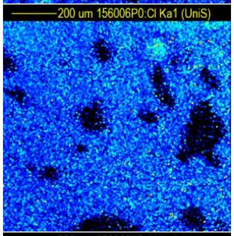
	**Fast Scanning**	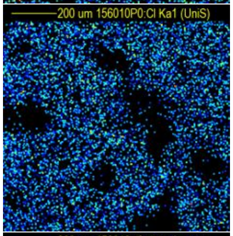	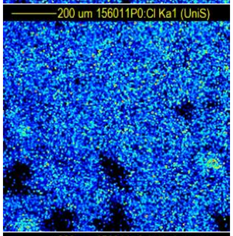	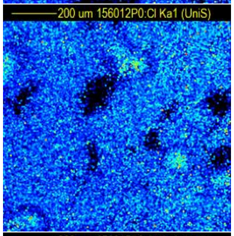
	**Random Scanning**	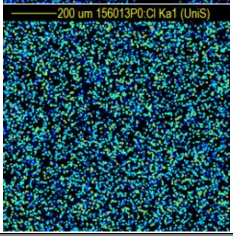	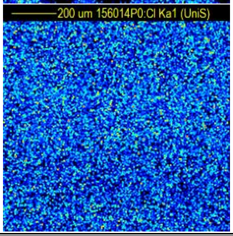	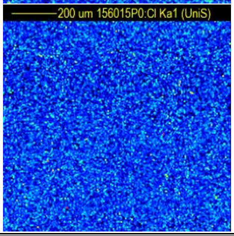
**Potassium (K)**	**Slow Scanning**	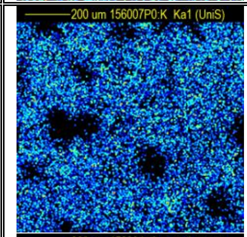	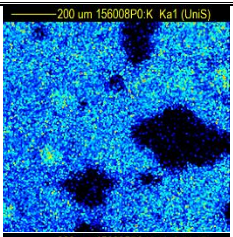	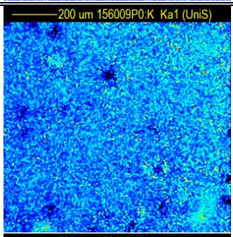
	**Standard Scanning**	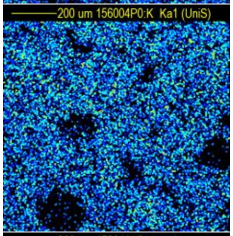	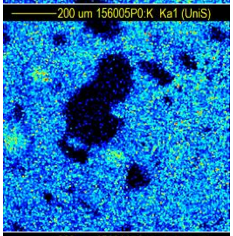	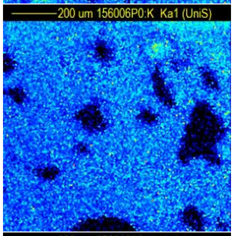
	**Fast Scanning**	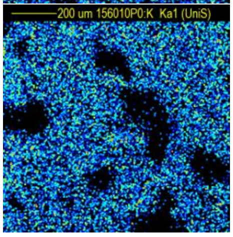	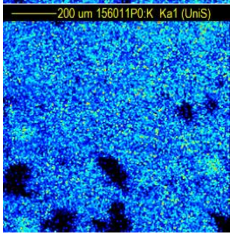	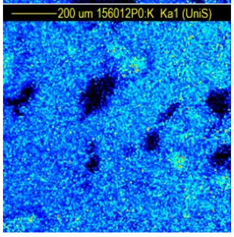

	**Random Scanning**	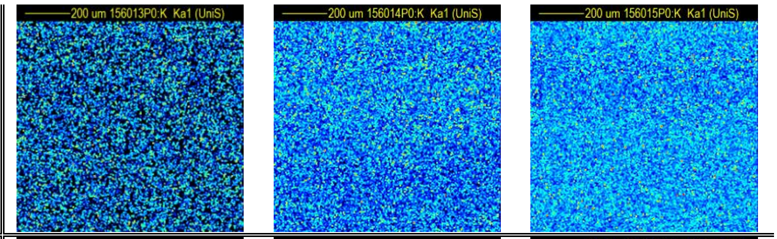
**Iron (Fe)**	**Slow Scanning**	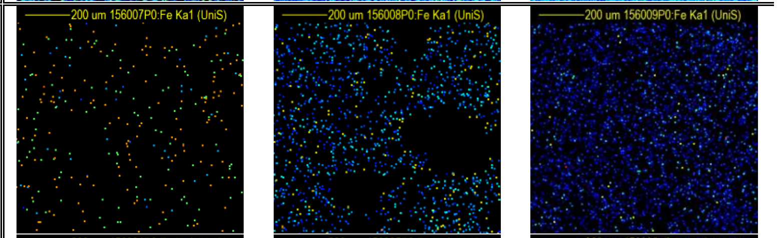
	**Standard Scanning**	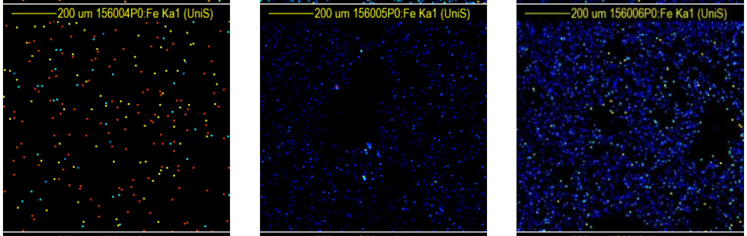
	**Fast Scanning**	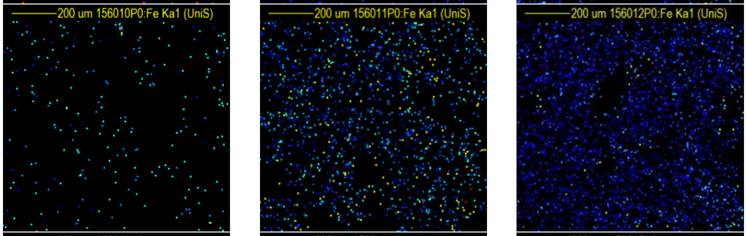
	**Random Scanning**	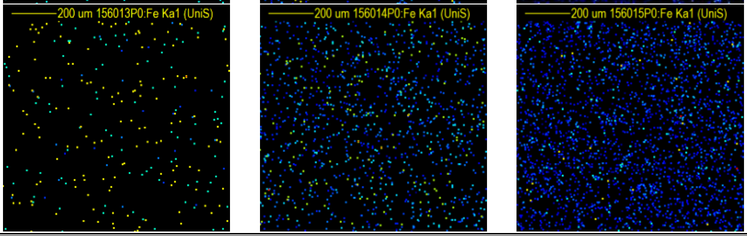

Figure S 4. Resulting PIXE maps (normalised to the total backscattered map) for phosphorus (P), sulphur (S), chlorine (Cl), potassium (K) and iron (Fe) at different fluences and scan speeds and pattern.

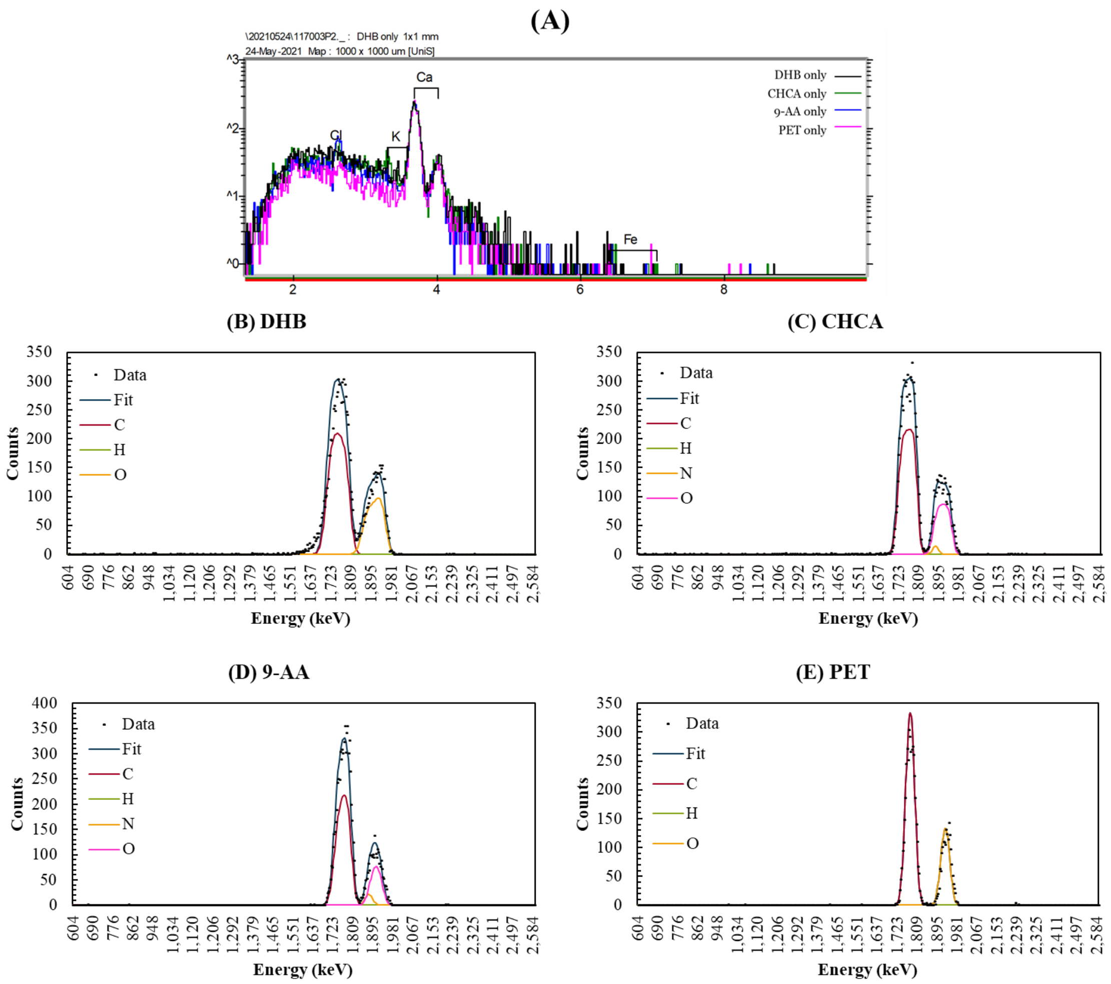

Figure S 5. (A) Overlay of X-ray and (B) fitted backscattered spectra taken from matrix-only areas (B-D). PET-only area (E) refers to the substrate. EBS spectra were fitted using OMDAQ-3.

Element	Matrix	Low Fluence	Medium Fluence	High Fluence
Phosphorus (P)	No Matrix	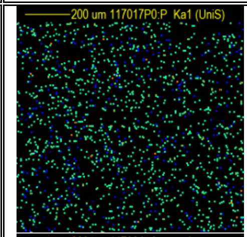	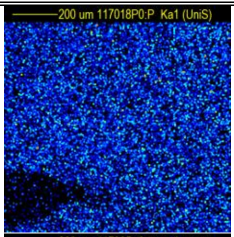	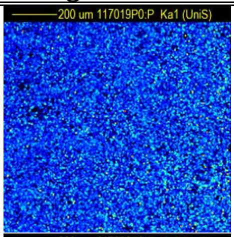
	DHB	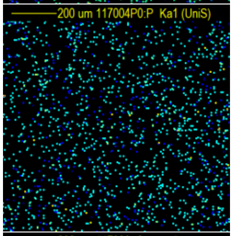	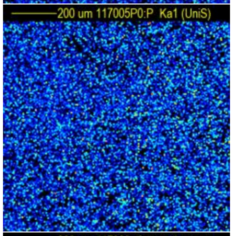	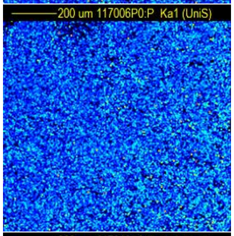
	CHCA	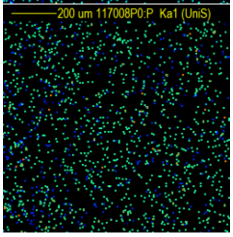	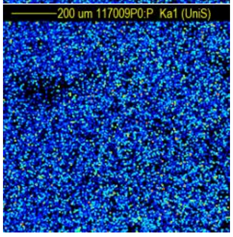	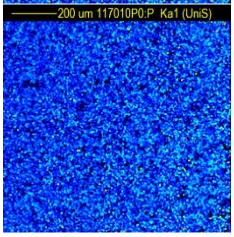

9-AA

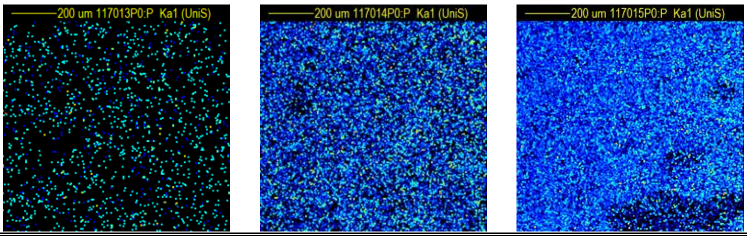

Sulphur (S)

No Matrix

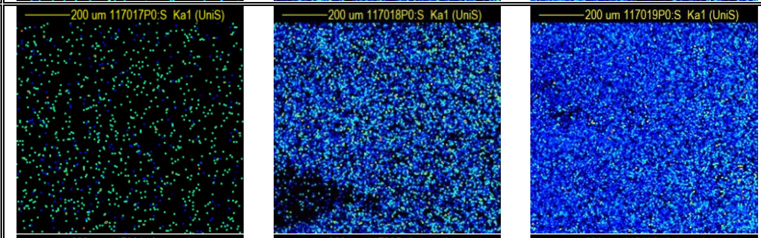

DHB

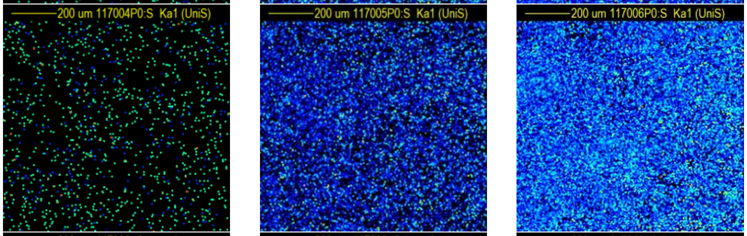

CHCA

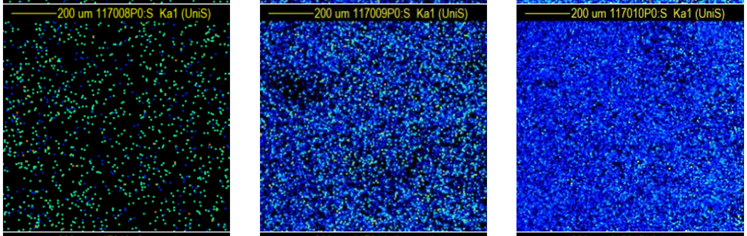

9-AA

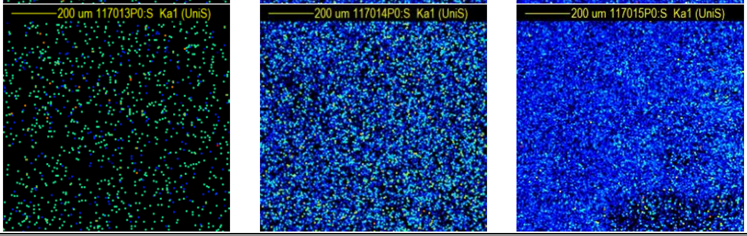

Chlorine (Cl)

No Matrix

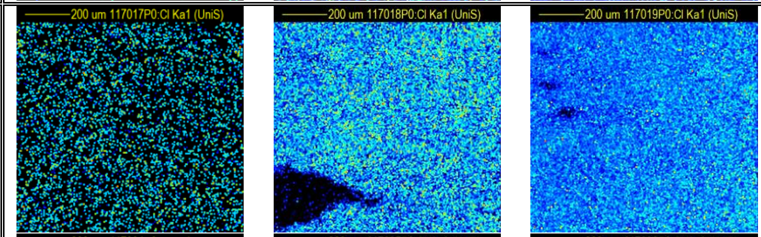

DHB

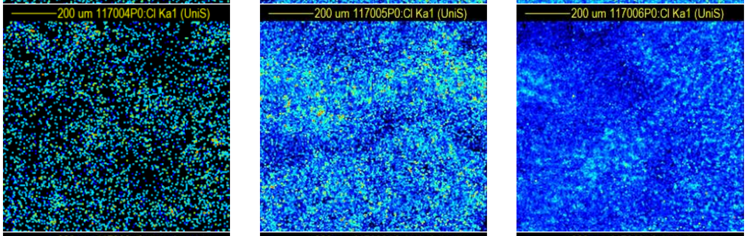

CHCA

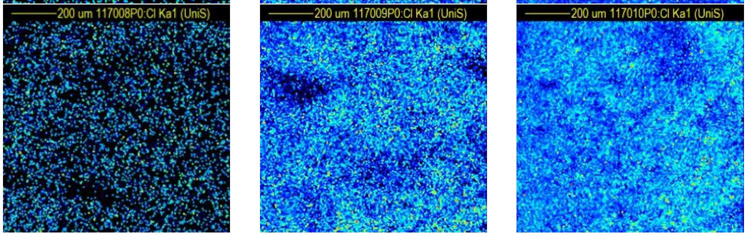

	9-AA
Potassium (K)	No Matrix
	DHB
	CHCA
	9-AA
Iron (Fe)	No Matrix
	DHB
	CHCA

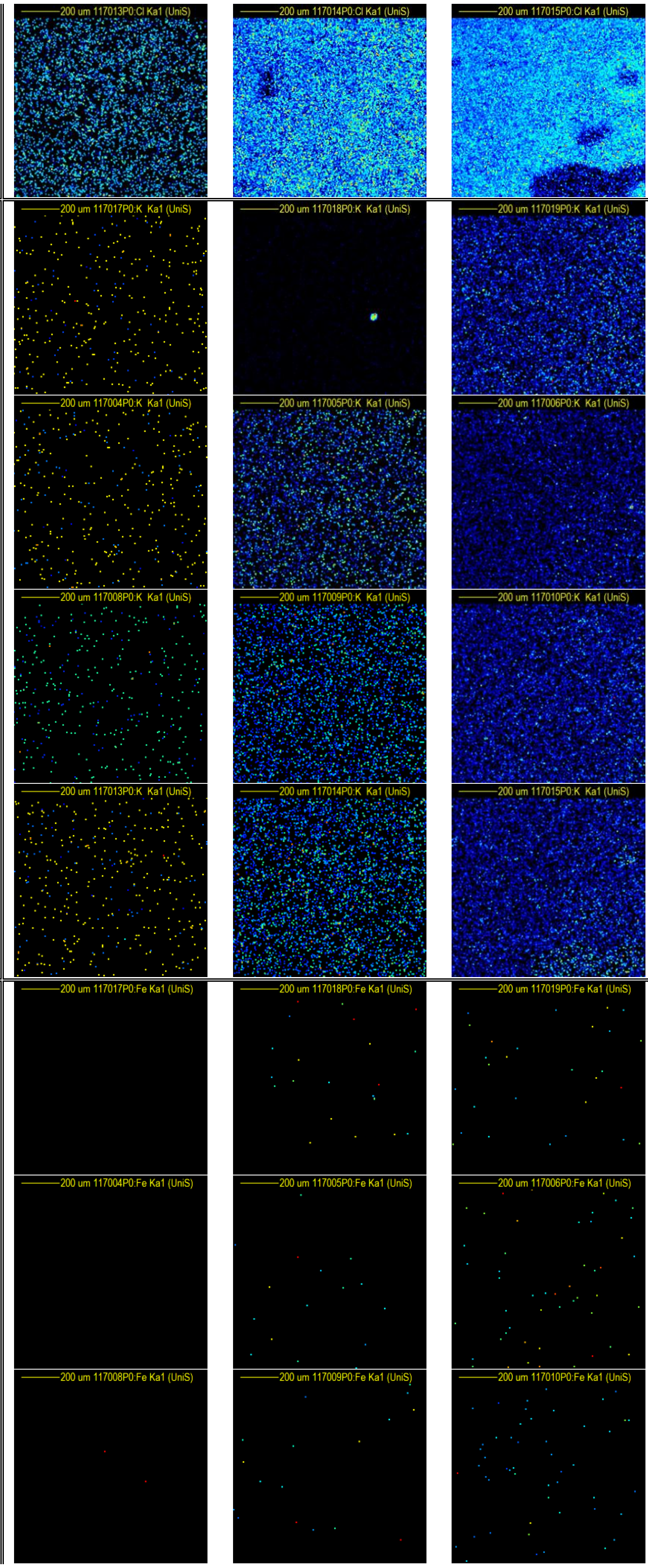

	**9-AA**	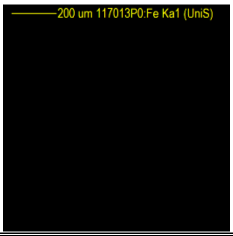	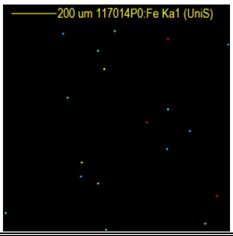	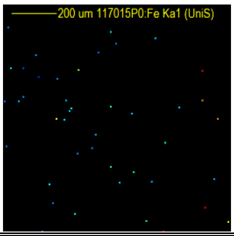

Figure S 6. Resulting PIXE maps (normalised to the total backscattered map) for phosphorus (P), sulphur (S), chlorine (Cl), potassium (K) and iron (Fe) at different fluences and in the presence/absence of a matrix.

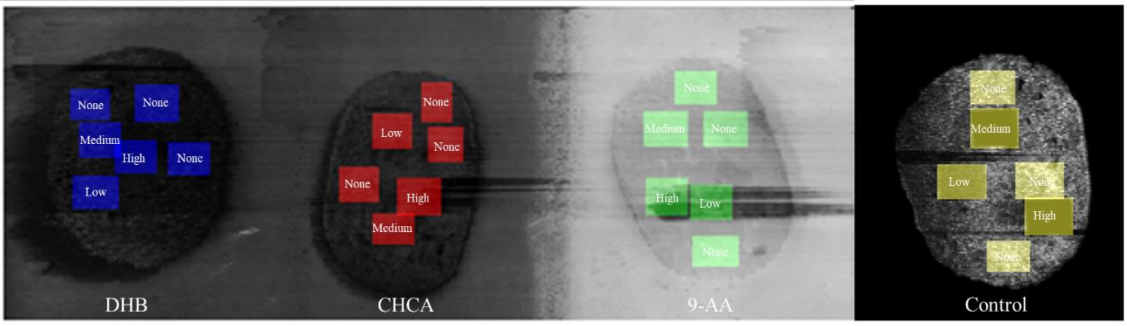

Figure S 7. Irradiated and non-irradiated areas (selected for data analysis) in liver tissue homogenates in the presence of DHB, CHCA, 9-AA and no matrix.

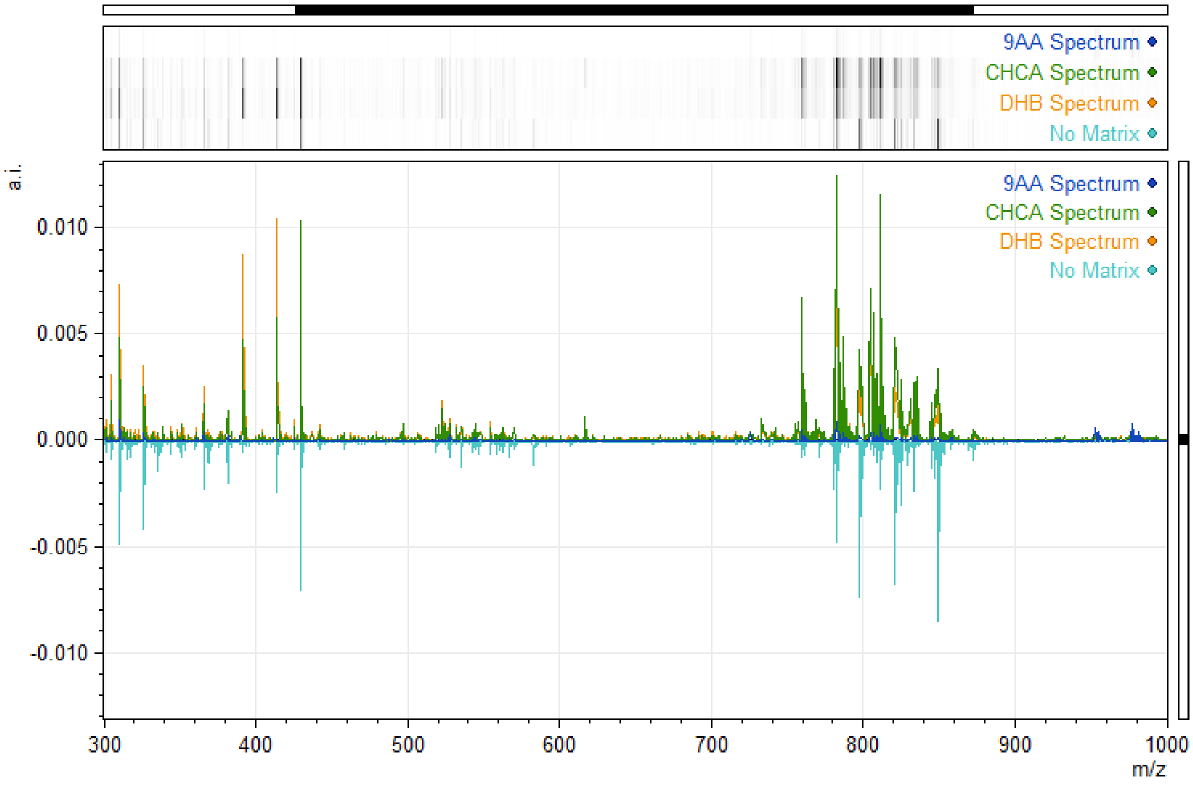

Figure S 8. DESI spectra overlay of liver homogenate tissue section regions (non-irradiated) coated with the different matrices and no matrix (flipped spectrum).

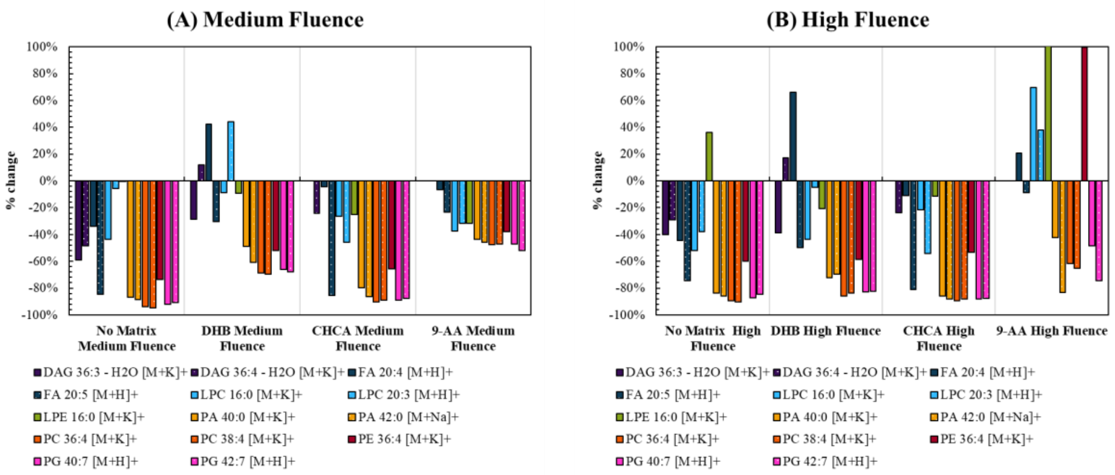

Figure S 9. % change in normalised (to TIC) peak intensity between ‘medium’ fluence (A) or ‘high’ fluence (B) and ‘no fluence’ irradiation ROIs on tissue sections coated with different matrices.

Table S 4. T-test results for the different lipid peaks observed in a control tissue section (no matrix) and a DHB-coated section, no matrix and a CHCA-coated section and no matrix and a 9-AA coated section. PND = peak not detected

No Matrix vs DHB

	Discovery?	P value	Mean of No Matrix	Mean of DHB	Difference	SE of difference	t ratio	df	q value
**DAG 36:3 - H2O [M+K]+**	**No**	**0.101192**	**4.33E-05**	**5.33E-05**	**-1.00E-05**	**4.71E-06**	**2.121**	**4**	**0.036501**
**DAG 36:4 - H2O [M+K]+**			**4.00E-05**	**4.00E-05**	**0**	**0**			
**FA 20:4 [M+H]+**	**Yes**	**0.001579**	**0.0003333**	**0.0005133**	**-0.00018**	**2.36E-05**	**7.637**	**4**	**0.000997**
**FA 20:5 [M+H]+**	**Yes**	**0.002915**	**0.0006433**	**0.0002733**	**0.00037**	**5.71E-05**	**6.485**	**4**	**0.001636**
**LPC 16:0 [M+K]+**	**Yes**	**0.0014**	**0.001157**	**0.0005833**	**0.0005733**	**7.27E-05**	**7.884**	**4**	**0.000997**
**LPC 20:3 [M+H]+**	**Yes**	**0.000282**	**0.0007867**	**0.00026**	**0.0005267**	**4.41E-05**	**11.94**	**4**	**0.000355**
**LPE 16:0 [M+K]+**	**Yes**	**0.000089**	**8.33E-05**	**0.00019**	**-0.00011**	**6.67E-06**	**16**	**4**	**0.00018**
**PA 40:0 [M+K]+**	**No**	**0.049693**	**0.002173**	**0.001723**	**0.00045**	**0.0001617**	**2.782**	**4**	**0.019304**
**PA 42:0 [M+Na]+**	**Yes**	**0.000032**	**0.0009567**	**0.0058**	**-0.004843**	**0.0002331**	**20.78**	**4**	**0.00008**
**PC 36:4 [M+K]+**	**Yes**	**0.009057**	**0.01236**	**0.008187**	**0.004173**	**0.000881**	**4.737**	**4**	**0.004158**
**PC 38:4 [M+K]+**	**Yes**	**0.000412**	**0.01132**	**0.003147**	**0.008173**	**0.0007544**	**10.83**	**4**	**0.000416**
**PE 36:4 [M+K]+**	**No**	**0.456153**	**0.000427**	**0.000447**	**-0.00002**	**2.43E-05**	**0.8242**	**4**	**0.162605**
**PG 40:7 [M+H]+**	**Yes**	**0.011608**	**0.005523**	**0.003817**	**0.001707**	**0.0003871**	**4.409**	**4**	**0.004885**
**PG 42:7 [M+H]+**	**Yes**	**0.004242**	**0.002813**	**0.001623**	**0.00119**	**0.0002032**	**5.856**	**4**	**0.002142**
**No Matrix vs CHCA**									
	**Discovery?**	**P value**	**Mean of No Matrix**	**Mean of CHCA**	**Difference**	**SE of difference**	**t ratio**	**df**	**q value**
**DAG 36:3 - H2O [M+K]+**			**4.33E-05**	**PND**					
**DAG 36:4 - H2O [M+K]+**	**No**	**0.116117**	**4.00E-05**	**3.33E-05**	**6.67E-06**	**3.33E-06**	**2**	**4**	**0.036085**
**FA 20:4 [M+H]+**	**Yes**	**0.000898**	**0.0003333**	**0.00052**	**-0.0001867**	**2.11E-05**	**8.854**	**4**	**0.000403**
**FA 20:5 [M+H]+**	**No**	**0.239848**	**0.0006433**	**0.00054**	**0.0001033**	**7.49E-05**	**1.379**	**4**	**0.069213**
**LPC 16:0 [M+K]+**	**Yes**	**0.000144**	**0.001157**	**0.0001667**	**0.00099**	**6.99E-05**	**14.16**	**4**	**0.000269**
**LPC 20:3 [M+H]+**	**Yes**	**0.00015**	**0.0007867**	**0.0001667**	**0.00062**	**4.42E-05**	**14.02**	**4**	**0.000269**
**LPE 16:0 [M+K]+**	**Yes**	**0.00749**	**0.00008333**	**0.0001**	**-0.00001667**	**0.000003333**	**5**	**4**	**0.002328**

**PA 40:0 [M+K]+**	**Yes**	**0.000538**	**0.002173**	**0.0006733**	**0.0015**	**0.0001483**	**10.12**	**4**	**0.000271**
**PA 42:0 [M+Na]+**	**Yes**	**0.006021**	**0.0009567**	**0.00139**	**-0.0004333**	**8.15E-05**	**5.316**	**4**	**0.002211**
**PC 36:4 [M+K]+**	**Yes**	**0.000293**	**0.01236**	**0.00281**	**0.00955**	**0.0008076**	**11.83**	**4**	**0.000269**
**PC 38:4 [M+K]+**	**Yes**	**0.000268**	**0.01132**	**0.002237**	**0.009083**	**0.0007513**	**12.09**	**4**	**0.000269**
**PE 36:4 [M+K]+**	**Yes**	**0.001959**	**0.0004267**	**0.0002567**	**0.00017**	**0.00002357**	**7.212**	**4**	**0.00072**
**PG 40:7 [M+H]+**	**Yes**	**0.000487**	**0.005523**	**0.001847**	**0.003677**	**0.0003543**	**10.38**	**4**	**0.000271**
**PG 42:7 [M+H]+**	**Yes**	**0.000469**	**0.002813**	**0.0009833**	**0.00183**	**0.0001746**	**10.48**	**4**	**0.000271**
**No Matrix vs 9-AA**									
	**Discovery?**	**P value**	**Mean of No Matrix**	**Mean of 9-AA**	**Difference**	**SE of difference**	**t ratio**	**df**	**q value**
**DAG 36:3 - H2O [M+K]+**			**4.33E-05**	**PND**					
**DAG 36:4 - H2O [M+K]+**			**4.00E-05**	**PND**					
**FA 20:4 [M+H]+**	**Yes**	**0.000109**	**0.0003333**	**3.33E-05**	**0.0003**	**1.97E-05**	**15.21**	**4**	**0.000188**
**FA 20:5 [M+H]+**	**Yes**	**0.000335**	**0.0006433**	**2.00E-05**	**0.0006233**	**5.46E-05**	**11.42**	**4**	**0.000395**
**LPC 16:0 [M+K]+**	**Yes**	**0.000101**	**0.001157**	**7.00E-05**	**0.001087**	**7.01E-05**	**15.51**	**4**	**0.000188**
**LPC 20:3 [M+H]+**	**Yes**	**0.000067**	**0.0007867**	**3.33E-05**	**0.0007533**	**4.38E-05**	**17.18**	**4**	**0.000188**
**LPE 16:0 [M+K]+**	**Yes**	**0.000025**	**0.00008333**	**0.00001**	**0.00007333**	**0.000003333**	**22**	**4**	**0.000176**
**PA 40:0 [M+K]+**	**Yes**	**0.000146**	**0.002173**	**9.33E-05**	**0.00208**	**0.0001474**	**14.11**	**4**	**0.000207**
**PA 42:0 [M+Na]+**	**Yes**	**0.000919**	**0.0009567**	**0.00044**	**0.0005167**	**5.87E-05**	**8.803**	**4**	**0.000928**
**PC 36:4 [M+K]+**	**Yes**	**0.000113**	**0.01236**	**0.00018**	**0.01218**	**0.0008074**	**15.09**	**4**	**0.000188**
**PC 38:4 [M+K]+**	**Yes**	**0.00012**	**0.01132**	**0.0001767**	**0.01114**	**0.0007506**	**14.85**	**4**	**0.000188**
**PE 36:4 [M+K]+**	**Yes**	**0.000058**	**0.0004267**	**0.000006667**	**0.00042**	**0.00002357**	**17.82**	**4**	**0.000176**
**PG 40:7 [M+H]+**	**Yes**	**0.000108**	**0.005523**	**0.00018**	**0.005343**	**0.0003506**	**15.24**	**4**	**0.000188**
**PG 42:7 [M+H]+**	**Yes**	**0.000091**	**0.002813**	**9.33E-05**	**0.00272**	**0.0001707**	**15.93**	**4**	**0.000188**

Table S 5. T-Test results comparing the % changes measured at medium fluence for the ambient irradiation and irradiation in the presence of DHB matrix experiments.

t-Test: Two-Sample Assuming Unequal Variances		
	Variable 1	*Variable 2*
Mean	0.637393225	0.472557
Variance	0.032438696	0.048173
Observations	18	18
Hypothesized Mean Difference	0	
df	33	
t Stat	2.463153146	
P(T<=t) one-tail	0.009579302	
t Critical one-tail	1.692360309	
P(T<=t) two-tail	0.019158605	
t Critical two-tail	2.034515297	
Conclusion: if P < 0.05, the difference between means is statistically significant.		
